# Genetic Testing and Pregnancy Outcome Analysis of 362 Fetuses with Congenital Heart Disease Identified by Prenatal Ultrasound

**DOI:** 10.5935/abc.20180144

**Published:** 2018-10

**Authors:** Shiyu Luo, Dahua Meng, Qifei Li, Xuehua Hu, Yuhua Chen, Chun He, Bobo Xie, Shangyang She, Yingfeng Li, Chunyun Fu

**Affiliations:** Maternal and Child Health Hospital of Guangxi Zhuang Autonomous Region, Guangxi - China

**Keywords:** Heart Defects, Congenital, Chromosome Disorders, Spectral Karyotyping, Pregnancy, Fetus, Ultrasonography

## Abstract

**Background:**

Congenital heart defects (CHD), as the most common congenital anomaly, have been reported to be associated with chromosomal abnormalities. Currently, patients with CHD are routinely offered karyotyping and chromosomal microarray (CMA) testing, but the genotype-phenotype relationship has not yet been fully established.

**Objective:**

To determine the type and frequency of chromosomal abnormalities in fetuses with CHD and to analyze pregnancy outcomes of fetuses with heart abnormalities caused by different genetic factors.

**Methods:**

A total of 362 cases of CHD were enrolled from 2009 to 2016. Detailed ultrasound and laboratory examinations, including karyotyping and CMA, were performed. Outcome was obtained from discharge summaries.

**Results:**

Of the 362 fetuses, 220 were found with an isolated CHD, and 142 had CHD with extracardiac anomaly. Among these 362 fetuses, 140 were identified with a genetic cause, including 111 cases with aneuploidy, 10 cases with abnormality of chromosomal structure by karyotyping and 19 cases with pathogenic or likely pathogenic copy-number variations (CNVs) by CMA. The detection rate is close to 38.7%. Only one (identified as trisomy 18 syndrome) in 140 positive cases resulted in perinatal death, with the others being induced. The remaining 222 cases had negative results for both genetic testing and of these cases, 56 resulted in induced labor, and 77 had natural childbirth or caesarean births. The pregnancy outcome of the remaining 89 cases was uncertain.

**Conclusions:**

Karyotyping and CMA are effective and accurate prenatal genetic techniques for identifying fetal chromosomal abnormalities associated with cardiac defects, and this can assist clinical doctors to perform appropriate genetic counselling with regard to the etiology and outcome of CHD.

## Introduction

Congenital heart disease (CHD), one of the most common birth defects, affecting approximately 1 in 100 live births.^1^^-^^3^ With the availability of advanced surgical techniques, normal or near normal cardiac function can be restored after surgical treatment of most types of CHDs ranging from simple ventricular septal defects (VSD) to more complex cardiovascular abnormalities. However, the long-term prognosis of a small, but significant number of CHD fetuses is usually complicated by severe extracardiac abnormalities, such as developmental delay and mental retardation. There is increasing evidence that genetic factors influence the development of most types of CHD,^4^^-^^6^ but the precise genetic basis of most CHD cases remains not fully understood. Current ultrasound technologies are able to detect most of CHD. However, it is difficult for physicians to make a comprehensive assessment of fetuses with CHD merely based on the evidence of prenatal ultrasound, as well as to manage the course of established pregnancy.^7^ Therefore, genetic testing is now highly recommended for fetuses with CHD.

Karyotyping has been the mainstream diagnostic method for detecting chromosomal abnormalities associated with CHD.^8^ For CHD cases in prenatal diagnosis, chromosomal anomalies are estimated to be as high as 22.^9^^,^^10^ Now, chromosomal microarray (CMA) has become the first tier technique in fetal structural anomalies detected by ultrasonography.^11^^,^^12^ The advent of CMA technology has allowed genome-wide searches of submicroscopic chromosomal deletions or duplications in the genome, known as copy-number variations (CNVs). CNV is a form of structural variation in the genome: specifically, it is a type of duplication or deletion that has an influence in the base pairs,^13^ and CNVs play an important role in generating necessary variation in the population and disease phenotypes.^14^ Recent studies have shown that a substantial proportion of CHD patients were detected with pathogenic CNVs,^15^^, ^^16^ and the syndromic or isolated CHD patients were found with multiple recurrent CNV loci, such as 22q11.2 (the DiGeorge syndrome region), 7q11.23, 8p23.1, 9q34.3, and 1q21.1.^17^^-^^19^

At present, only a few studies have reported genetic testing among large groups of fetuses with CHD in China,^20^^-^^24^ the genotype-phenotype relationship has not yet been fully established. The Laboratory of Genetics and Metabolism from the Maternal and Child Health Hospital in Guangxi is one of the largest Perinatal Diagnostic centers in South China. This study aimed to analyze the chromosomal abnormalities and pregnancy outcomes in 362 fetuses with CHD.

## Methods

### Subjects

Fetal ultrasound anatomy scans were routinely performed for pregnant women at the Prenatal Diagnosis Center of Guangxi Zhuang Autonomous Region in China. The anatomy scans were conducted between 20 and 28 weeks of gestation by senior sonographers using GE E8 ultrasound machines (General Electric Healthcare, USA). If CHD was suspected, the echocardiography was subsequently performed for confirmation.

A total of 8,430 pregnancies between June 2012 and June 2016 were screened for fetal cardiac defects, and 362 fetuses were identified with CHD. The Medical Ethics Committee of the Guangxi Maternal and Child Health Hospital approved the study protocol (Approval no.160220), and the parents of all selected fetuses with CHD gave their written consent.

### Testing of SNP microarray

All samples of amniotic fluid or fetal cord blood were collected from the pregnant women, and genomic DNA was extracted using the QIAamp DNA Blood Mini Kit (Qiagen, Germany) according to the manufacturer's protocol. SNP (Single Nucleotide Polymorphism) microarray testing was performed using Illumina HumanCytoSNP-12 v2.1 BeadChip (Illumina, USA). The laboratory policy at the time of testing was not to report well-established polymorphisms, CNVs that do not contain genes and CNVs smaller than 0.20 Mb. However, stretches of homozygosity larger than 10 Mb were reported.

### Karyotyping

All samples of amniotic fluid or fetal cord blood were used to perform G-banding according to the standard procedure as described previously.^25^

## Results

### Clinical data

Among the 8,430 pregnancies, 362 cases of CHD were diagnosed using fetal echocardiography, for a frequency of 4.2%. The mean age of the pregnant women was 31.1 ± 5.1 years, and the mean gestational week at diagnosis was 24.4 ± 3.8 weeks.

The 5 most common types of CHD were, in order, ventricular septal defect (51.9%, 188/362), persistent left superior vena cava (13.0%, 47/362), endocardial cushion defects (0.9%, 33/362), single umbilical artery (0.9%, 32/362) and right-sided aortic arch (0.8%, 29/362).

### Etiology

In total, 362 fetuses were diagnosed with CHD. The genetic tests found 111 cases with aneuploidy, 10 cases with abnormality of chromosome structure, and 19 cases with pathogenic or likely pathogenic CNVs (Table 1). The remaining 222 cases showed no abnormal genetic findings. The abnormalities of chromosome numbers consisted of trisomy 18 syndrome (61 cases), trisomy 21 syndrome (31 cases) and trisomy 13 syndrome (19 cases). CMA identified 19 CNVs, including DiGeorge syndrome (8 cases), Jacobsen syndrome (2 cases), Angelman/Prader-Willi syndrome (1 case), 16p11.2-p12.2 microdeletion syndrome (1 case), 16q24-triplication syndrome (1 case), Thrombocytopenia-absent radius (TAR) syndrome (1 case), 3q29 microduplication syndrome (1 case), 22q11 duplication syndrome (1 case), Cri du chat syndrome (1 case) and 2 likely pathogenic CNVs (Table 2).

**Table 1:** Genetic testing of 362 fetuses with congenital heart defects

Etiology	Classifications	Numbers
Aneuploidyn(111, 30.7%)	Trisomy 18	61
	Trisomy 21	31
	Trisomy 13	19
Abnormality of chromosome structure (10, 2.8%)	46,X,i(X)(q10)	1
	46,der(18)dup(18)(q11q22)del(18)(q22q23)	1
	46,XY,r(13)(p13q34)	1
	46,XY,der(21;21)(q10;q10),+21	1
	46,XX,der(9)t(9;18)(p22;q21)mat	1
	46,XY,del(10)(q11q22)dn	1
	46,XY,6q-dn	1
	46,XY,der(18)t(7;18)(q22;q23)mat	1
	46,XX,del(5)(p13)	1
	46,XY,der(5)t(5;12)(p13;p12)mat	1
CNVs (19, 5.2%)	15q13.2q13.3(30940398-32515681)x1	1
	arr16p11.2(29614976-30199805)x1~2	1
	arr16q21q24.3(63,863,382-90,130,136)x2~3	1
	arr1q21.1q21.2(146,501,348-147,828,939)x1	1
	arr3q21.1q29(123031042-198022430)x2~3	1
	arr22q11.21(18877787-21458625)x1	1
	arr22q11.21(18889490-21460220)x1	1
	arr22q11.21(18895703-21928916)x1	1
	arr 22q11.21(18844632-21462353)x1	1
	arr 11q24.1q25(123615329-1349444006)x1	1
	arr10q26.13q26.3 (126254468-135430043)x3, arr11q24.1q25(122805910-134944006)x1	1
	arr 10p15.1p12.31(6085312-21544231)x1	1
	arr 5q11.2q12.1(56368573-61428613)x1	1
	arr21q11.2 q21.1(14687571-18341062)x1	1
	arr22q11.21(21050552-21811991)x1	1
	arr22q11.21(20740778-21445064)x1	1
	arr22q11.21(18895703-21452237)x1	1
	arr11q23.3q25(116728277-134944006)x3, arr22q11.1q11.21(16079545-20306993)x3	1
	arr5p15.33p15.1(354051-17484038)x1, 5q34q35.3(165731079-180705539)x3	1

*CNVs: copy-number variations.*

**Table 2:** Copy-number variations (CNVs) in 362 fetuses with congenital heart disease (CHD)

Patient	Cardiac defect	Extra-cardiac defect	CNVs	Size (Mb)	Known syndrome/candidate genes related to CHD	Classification
1	persistent left superior vena cava	Intrauterine growth retardation	15q13.2q13.3(30940398-32515681)x1	10.0	Angelman/Prader-Willi syndrome	pathogenic
2	persistent left superior vena cava, single umbilical artery		arr16p11.2(29614976-30199805)x1~2	0.5	16p11.2-p12.2 microdeletion syndrome	pathogenic
3	pulmonary stenosis		arr16q21q24.3(63,863,382-90,130,136)x2~3	26.3	16q24-triplication syndrome	pathogenic
4	complete-type endocardial cushion defect		arr1q21.1q21.2(146,501,348-147,828,939)x1	1.3	Thrombocytopenia-absent radius (TAR) syndrome	pathogenic
5	ventricular septal defect	Short limbs	arr3q21.1q29(123031042-198022430)x2~3	75.0	3q29 microduplication syndrome	pathogenic
6	tetralogy of fallot		arr22q11.21(18877787-21458625)x1	2.6	DiGeorge syndrome	pathogenic
7	tetralogy of fallot		arr22q11.21(18889490-21460220)x1	2.6	DiGeorge syndrome	pathogenic
8	right aortic arch, persistent left superior vena cava		arr22q11.21(18895703-21928916)x1	3.0	DiGeorge syndrome	pathogenic
9	tetralogy of fallot, absent pulmonary valve		arr 22q11.21(18844632-21462353)x1	2.6	DiGeorge syndrome	pathogenic
10	single umbilical artery		arr 11q24.1q25(123615329-1349444006)x1	11.3	Jacobsen syndrome	pathogenic
11	endocardial cushion defect, single atrium		arr10q26.13q26.3 (126254468-135430043)x3, arr11q24.1q25(122805910-134944006)x1	9.2; 12.1	Jacobsen syndrome	pathogenic
12	Atria septal defect		arr 10p15.1p12.31(6085312-21544231)x1	15	CACNB2	likely pathogenic
13	ventricular septal defect		arr 5q11.2q12.1(56368573-61428613)x1	5.1		likely pathogenic
14	ventricular septal defect, atrial septal defect		arr21q11.2q21.1(14687571-18341062)x1	3.7	DiGeorge syndrome	pathogenic
15	ventricular septal defect	fetal cystic hygroma	arr22q11.21(21050552-21811991)x1	0.7	DiGeorge syndrome	pathogenic
16	ventricular septal defect		arr22q11.21(20740778-21445064)x1	0.7	DiGeorge syndrome	pathogenic
17	tetralogy of fallot, thymic hypoplasia	Intrauterine growth retardation	arr22q11.21(18895703-21452237)x1	2.6	DiGeorge syndrome	pathogenic
18	pulmonary valve stenosis, aortic coarctation ventricular septal defect		arr11q23.3q25(116728277-134944006)x3; arr22q11. 1q11.21(16079545-20306993)x3	18	22q11 duplication syndrome	pathogenic
19	ventricular septal defect, Small left heart	Intrauterine growth retardation	arr5p15.33p15.1(354051-17484038) x1,5q34q35.3(165731079-180705539)x3	17.1; 15.0	Cri du chat syndrome	pathogenic

### Occurrence of fetal cardiac malformations

Of the 362 CHD, 181 fetuses were found with single cardiac malformations, and 181 were found with multiple cardiac abnormalities; 220 were found with an isolated CHD; and 142 had CHD with extracardiac anomaly. Table 3 lists the etiology of the various types of fetal cardiac malformations observed.

**Table 3:** Genetic detection in different categories of fetuses with congenital heart disease (CHD)

Classification of CHD	Aneuploidy	Abnormality of chromosome structure		CNVs	Other
Single cardiac malformation (n = 181)	40	4		9	128
Multiple cardiac abnormalities (n = 181)	71	6		10	94
Isolated CHD (n = 220)	26	8		14	172
CHD with extracardiac anomaly (n = 142)	85	2		5	50

*CNVs: copy-number variations.*

### Pregnancy Outcomes

**Figure 1:**
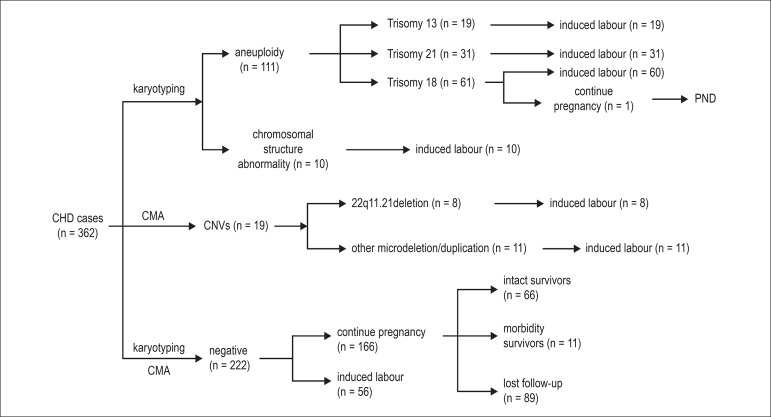
The patient pathway in the current study. PND: perinatal deaths. CMA: chromosomal microarray; CNVs: copy-number variations; CHD: congenital heart defects.

Among all 140 cases with a positive genetic testing result, only one woman chose to continue her pregnancy, and the rest of them chose to induce labor. The fetus was diagnosed with trisomy 18 syndrome, presenting difficulties in feeding, and died 4 days after birth. Among the remaining 222 negative cases, 56 were subjected to labor induction, and most of these cases were deemed incurable or had poor prognostic cardiac malformations (including single ventricle, left or right ventricular dysplasia and tetralogy of fallot) or were complicated with extracardiac anomalies (Figure 1).

Mothers of 77 fetuses with mild or curable cardiac malformations chose to maintain their pregnancies. Of these cases, 66 were found with no abnormality after birth, 8 cases needed surgery, one presented delayed development, one was found with clubfoot, one was identified with hypomyotonia, and the pregnancy outcomes of the remaining 89 cases were uncertain (Figure 1).

## Discussion

In this study, 362 cases of fetal CHD were identified in a total of 8,430 pregnancies at a single Maternal and Children's hospital from the Southern region of China from June 2012 to June 2016, with an incidence of 4.2%. This incidence was similar to that reported in Xi'an, in Northwestern China,^26^ and higher than the rate of 2.3% reported in Guangzhou, In southern China.^23^ Among the 362 CHD fetuses, ventricular septal defect (51.9%, 188/362) and persistent left superior vena cava (13.0%, 47/362) were the most prevalent cardiac abnormalities detected by ultrasound scans.

Many factors such as genetic factors (including chromosomal abnormalities and gene mutations) and risk factors associated with mothers (including the rubella virus, other infections, radiation, drug use and environmental pollution) are reported to be associated with CHD.^5^^-^^7^^,^^27^^-^^29^ However, the causes of most types of CHD are still poorly understood. In our study, 140 of 362 CHD fetuses were identified with clinically significant chromosomal abnormalities by karyotyping and CMA, with a detection rate of up to 38.7%. The positive rates of genetic testing in this study is far higher than previous reports in Chongqing, China^24^ and the Netherlands.^30^ This rate is similar to that of Brazilians.^31^

Among the 140 chromosomal abnormalities, 111 (79.3%) were aneuploidy, of which trisomy 18 was the most common; 10 (7.1%) cases were abnormality of chromosome structure; and 19 (13.6%) cases were pathogenic or likely pathogenic CNVs. It is suggested that aneuploidy is the leading genetic cause of fetuses with CHD in our population. Given that G-banding can only reliably detect structural abnormalities > 10 Mb in size, 11 pathogenic CNVs may be missed by karyotyping but detected by CMA. On this basis, we estimate that the incremental yield of reportable CNVs with less than 10 Mb achieved by CMA was 3.0%.

Complex multiple cardiac malformations have poor prognosis and heavily affect the quality of life of surviving infants, but cases such as mild tetralogy of fallot have a reasonable outcome after surgery, as well as a good prognosis. In our study, ultrasonic results of some fetuses with CHD caused by aneuploidy only displayed mild cardiac malformations, although complex CHD combined with extra cardiac defects were more common in these cases. Besides, some symptoms such as mental disability cannot be found by prenatal ultrasound. In these cases, the results of genetic testing is of great importance, because this situation is easily ignored by patients and clinicians. However, several negative cases featured complex CHD and extra cardiac defects after karyotype and CMA testing, and these cases provide an important clue for the study of other factors that lead to CHD.

Several limitations should be considered in the study when reviewing these findings. Firstly, a comprehensive analysis of all known CHD associated genes was not carried out. Secondly, the inheritance of CNVs in some cases with likely pathogenicity was not identified.

## Conclusion

Karyotyping and CMA analysis was conducted in 362 CHD fetuses, and it was found that 38.7% of CHD fetuses had a positive genetic testing result. Aneuploidy is the major cause of CHD fetuses in our population. The combination of ultrasonic detection and genetic testing can effectively diagnose fetuses with cardiac malformations and extra cardiac defects, thus providing valuable information to the clinician and patients.
